# Supplementary material for: Different Components of the RNA Interference Machinery Are Required for Conidiation, Ascosporogenesis, Virulence, Deoxynivalenol Production, and Fungal Inhibition by Exogenous Double-Stranded RNA in the Head Blight Pathogen Fusarium graminearum
Source: Front Microbiol. 2019 Aug 7;10:1662. doi: 10.3389/fmicb.2019.01662 (PMC6764512; doi:10.3389/fmicb.2019.01662)
Supplement: Supplementary file 1 [file Data_Sheet_1.pdf]

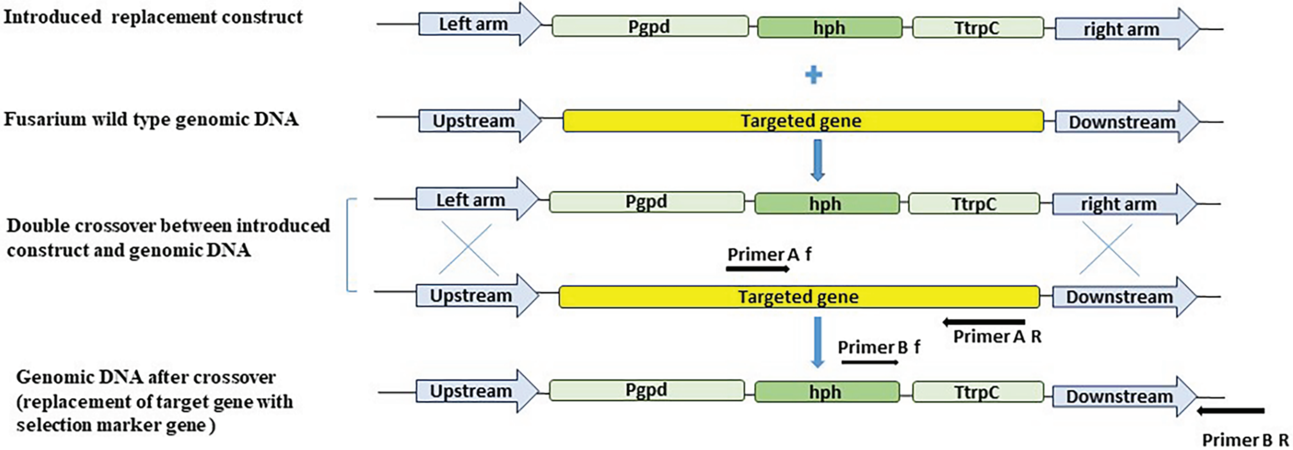

FIGURE S1. Schematic representation of the gene replacement strategy used for *F. graminearum* transformation. Yellow box: the target gene that has to be replaced by KO; dark green box: selection marker gene, in this case the antibiotic resistance gene (*Hygromycin B phosphotransferase* of *E. coli*, hph). Blue arrow: Homologous recombination sequences, typically ~1 kb long; Black arrows: template area for primers binding used for transformants genotyping. PgpdA: Promoter region of the *Glyceraldehyde-3-phosphate dehydrogenase* gene of *Aspergillus nidulans*; TtrpC: termination region of the *Aspergillus nidulans trpC* gene.

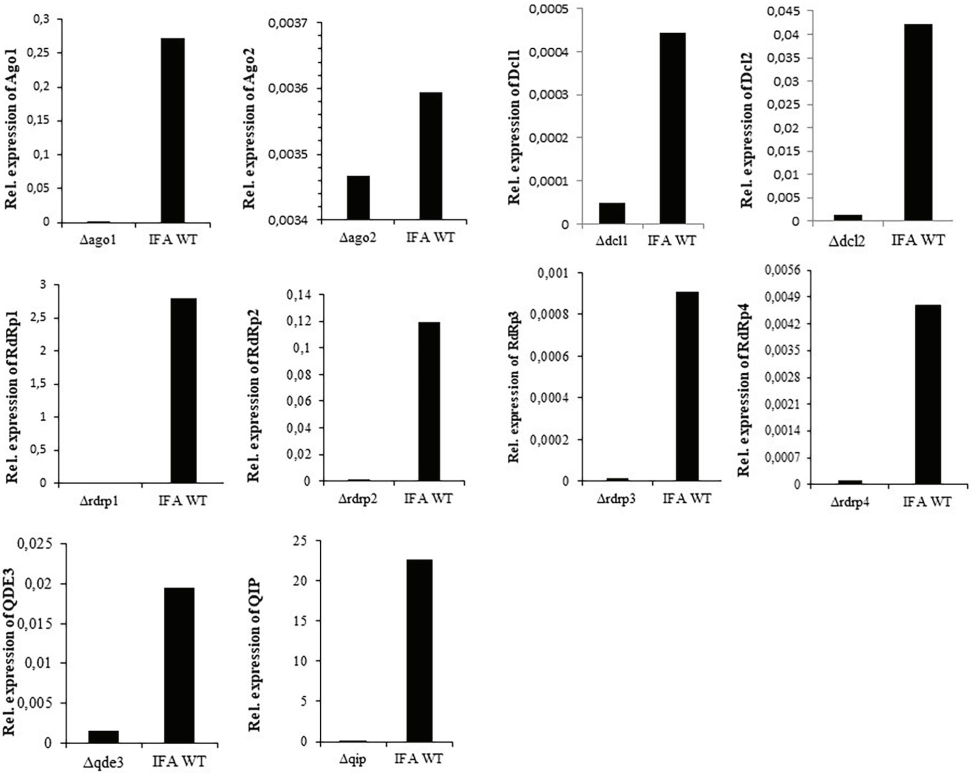

FIGURE S2. Compromised expression of deleted RNAi genes in *F. graminearum* knockout (KO) mutants. Expression of the targeted genes in respective *Fusarium* mutants. Transcript levels were analyzed by qRT-PCR from 5-day-old PEG liquid cultures and transcript quantified by normalization to *Fusarium* β*-Tubulin (FgTub)* or *Elongation factor A (FgEF1a)* and comparison to IFA WT.

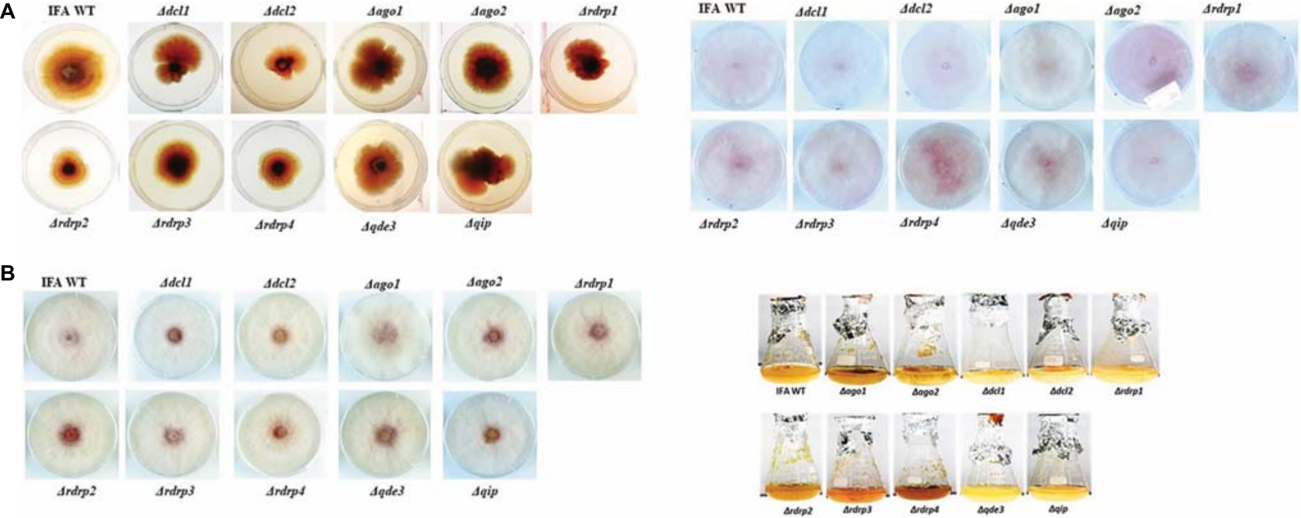

FIGURE S3 . Colony morphology and growth of RNAi KO mutants. Fusarium mutants and IFA WT were grown for 5 days on solid **(A)** PDA (potato dextrose agar), **(B)** SN (synthetic nutrient), **(C)** CM (Aspergillus complete medium) and **(D)** in liquid PEG medium without hygromycin. The mutants showed differences in pigmentation as follows: *Δago1*, *Δrdrp2*, *Δrdrp3* and *Δrdrp4* darker pigmentation; *Δdcl1*, *Δdcl2*, and *Δrdrp1* reduced pigmentation compared to IFA WT.

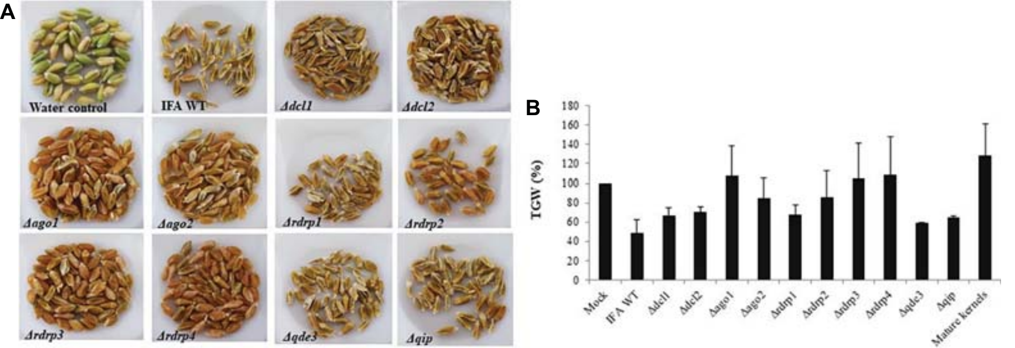

FIGURE S4. **(A)** Kernels from wheat spikes infected with *F. graminearum* RNAi mutants or IFA WT. **(B)** Thousand grain weight (TGW) of kernels from infected wheat spikes. Mock control: Kernels treated with 0.002% Tween 20; mature kernels: completely mature Apogee kernels.
