# Supplementary material for: Different Components of the RNA Interference Machinery Are Required for Conidiation, Ascosporogenesis, Virulence, Deoxynivalenol Production, and Fungal Inhibition by Exogenous Double-Stranded RNA in the Head Blight Pathogen Fusarium graminearum
Source: Front Microbiol. 2019 Aug 7;10:1662. doi: 10.3389/fmicb.2019.01662 (PMC6764512; doi:10.3389/fmicb.2019.01662)
Supplement: Supplementary file 2 [file Table_1.docx]

**Table S1**. Primers used for construction of disruption plasmids.

| AGO1PacI_F GCC**TTAATTAA**GCCCAAGGAGAGAAAACGCA  AGO1KpnI_R GTC**GGTACC**ACACTAACATCAACTTCGGC | Primers for amplifying upstream flanking fragment of *AGO1* |
| --- | --- |
| AGO1XbaI_F CGG**TCTAGA**TAGAAAGATTAATCGTATAA  AGO1HindIII_R CGC**AAGCTT**GCTGGCTTGACTATGCCCGT | Primers for amplifying downstream flanking fragment of *AGO1* |
| QDE-3PacI_F CGC**TTAATTAA**ATCACCTTCGCAGTCGAGTA  QDE-3KpnI_R GTC**GGTACC**GAAGCCGACGCAATTATACA | Primers for amplifying upstream flanking fragment of *QDE3* |
| QDE-3XnaI_F CCG**TCTAGA**GTAGGAGGAGAGATCAATTG  QDE-3HindIII_R GGC**AAGCTT**GACGAGCCTTGTTCCTGTTT | Primers for amplifying downstream flanking fragment of *QDE3* |
| QIPPacI_F CCG **TTAATTAA**TTGAGGATGCAACTAAGCAA  QIPKpnI_R CGC**GGTACC** CGCTGGTATTCTCTGCAAGGC | Primers for amplifying upstream flanking fragment of *QIP* |
| QIPXbaI_FCCG**TCTAGA**ATGATGAATCCTCGTTTGGGC  QIPHindIII_R CCC**AAGCTT**ATCGATAACAACCCCGGACAA | Primers for amplifying downstream flanking fragment of *QIP* |
| RDRP1PacI_F CCG**TTAATTAA**ATGCGCGATAGTGGGTGAGA  RDRP1KpnI_R GTC**GGTACC**GGCGACCGGCGCCAGTTGTG | Primers for amplifying upstream flanking fragment of *RdRP1* |
| RDRP1XbaI_F GCG**TCTAGA**GTGAGGTCTCTTAGAGGTTC  RDRP1HindIII_R GTC**AAGCTT**TTCAGTTGCTAACTACTTCA | Primers for amplifying downstream flanking fragment of *RdRP1* |
| DCL1PacI_F CTC**TTAATTAA** AAAACCTGAGGTTACCATGC  DCL1KpnI_R CTC **GGTACC** ATTGAGGCTGTATTTGTTCT | Primers for amplifying upstream flanking fragment of *DCL1* |
| DCL1XbaI_F CGG**TCTAGA**AGTACTGCAGGGTATTTAGT  DCL1HindIII_R CGG**AAGCTT**AATAAAAGACAACAACAGAAGG | Primers for amplifying downstream flanking fragment of *DCL1* |
| DCL2PacI_F CCG**TTAATTAA**CAAGGATAAAAGCTGATTGT  Dcl2KpnI_R GCC**GGTACC**CCTGATCTGCCGAAGCCCTC | Primers for amplifying upstream flanking fragment of *DCL2* |
| DCL2XbaI_F GCC**TCTAGA**GTTGGAGATTTCTCCTCGTA  DCL2HindIII_R GCC**AAGCTT**CTGGGACTGTGCCGAGGCCA | Primers for amplifying downstream flanking fragment of *DCL2* |
| AGO2PacI_F GCG **TTAATTAA**TCTCATCGAGGTCATTTCAT  AGO2KpnI_R CGC **GGTACC**CCAGAACAATGGCGTTGATG | Primers for amplifying upstream flanking fragment of *AGO2* |
| AGO2XbaI_F CGG **TCTAGA**TGGTGACAGATGAGTCTGAA  AGO2XbaI_R CGG **TCTAGA**GCTTTGATATAAACAATAAA | Primers for amplifying downstream flanking fragment of *AGO2* |
| RDRP4PacI_F TCGTTAATTAA CAGAGGTCGGGTAAAACTATCT  RDRP4KpnI_R CGTGGTACCATGTTCTTTATCCGGGTGGGCC | Primers for amplifying upstream flanking fragment of *RdRP4* |
| RDRP4XbaI_F CCGTCTAGATTGTTTCTTTTGTAAATTTCGTAG  RDRP4HindIII_R CGTAAGCTTTCCGGTGAGAAAACAACAGA | Primers for amplifying downstream flanking fragment of *RDRP4* |
| RDRP2PacI_F CGCTTAATTAAGGTGGTTCTTATACCTTCGTTC  RDRP2KpnI_R TCCGGTACCGCGATCTCTTCTCGTAGCG | Primers for amplifying upstream flanking fragment of *RdRP2* |
| RDR2XbaI_F CGCTCTAGATTTCCAGCTTCTATGAGTGTGCAGTTG  RDR2HindII_R GCCAAGCTTTCTCGGCGGATACTTGGCTGAT | Primers for amplifying downstream flanking fragment of *RdRP2* |
| RDR3PacI_F TCGTTAATTAA CCCAGGCTTGGGCTGCATAGTG  RDR3KpnI_R CGTGGTACC CGTTGAGGCAGGGCAAGACTGA | Primers for amplifying upstream flanking fragment of *RdRP3* |
| RDR3XbaI_F CGC TCTAGA AACCTTTCAGGTCACTGAGC  RDR3HindIII reverse:GCC AAGCTTAGTAGCCTGGTCGTTGCGGAAC | Primers for amplifying downstream flanking fragment of *RdRP3* |
