# Supplementary material for: Different Components of the RNA Interference Machinery Are Required for Conidiation, Ascosporogenesis, Virulence, Deoxynivalenol Production, and Fungal Inhibition by Exogenous Double-Stranded RNA in the Head Blight Pathogen Fusarium graminearum
Source: Front Microbiol. 2019 Aug 7;10:1662. doi: 10.3389/fmicb.2019.01662 (PMC6764512; doi:10.3389/fmicb.2019.01662)
Supplement: Supplementary file 3 [file Table_2.docx]

**Table S2.** Primers used for genotyping of the *Fg* transformants.

| DCL2_R* GTCTAACTGACCTAAAGTCCATGATTGAGA | Reverse primer located in *DCL2* downstream flanking sequence (used with hygromycin forward primer) |
| --- | --- |
| AGO1_R* ATGGCTTCCCAAAGGTAACCTAGGCTCGAC | Reverse primer located in *AGO1* downstream flanking sequence (used with hygromycin forward primer) |
| RDR1_R* ATTTCGCACCACGTATCGGCGTGACCTTAC | Reverse primer located in *RdRP1* downstream flanking sequence (used with hygromycin forward primer) |
| QDE3_R* CAGTCTTCTCCCTCAGCCCAGATCGGAAAC | Reverse primer located in *QDE3* downstream flanking sequence (used with hygromycin forward primer) |
| QIP_R* CCTCGATATCGTAGATGCTCGGCCTCCAAG | Reverse primer located in *QIP* downstream flanking sequence (used with hygromycin forward primer) |
| AGO2­_R* TATTCAAACAACGTATGATTTTCTTTTCTT | Reverse primer located in *AGO2* downstream flanking sequence (used with hygromycin forward primer) |
| DCL1DAR* AACATCCACGCTTGACAACCAAGATTCGGA | Reverse primer located in *DCL1* downstream flanking sequence (used with hygromycin forward primer) |
| HygB2*_F CACTCGTCCGAGGGCAAAGGAATAG | Forward Hygromycin primer |
| RDR4flank_R GTCACACACAGAAGGTTGAACCAGTAGCTG | Reverse primer located in *RdRP4* downstream flanking sequence (used with hygromycin forward primer) |
| RDR2flank_R TGCGCTCGATATGGATGGAACTTGCACGGG | Reverse primer located in *RdRP2* downstream flanking sequence (used with hygromycin forward primer) |
| RDR3flank_R GCTTATGTTAACAAACCACGATTCTGTATA | Reverse primer located in *RDR3* downstream flanking sequence (used with hygromycin forward primer) |
| RDR2G_F CAATCTCCCATCTCTCTGAATAAC  RDR2G_R CATCGCGAGTAGTGGAAAAACACTG | Primers amplifying part of *RdRP2* |
| RDR3G_F ATGGAAGTCATCTGCCGAAATGTC  RDR3G_R AGGCTTGACCAATACGTGCTGCAC | Primers amplifying part of *RdRP3* |
| RDR4Gf_F CAGTACACTCCAGCTTGGGAAGACC  RDR4G_R CCAGGATTCGTTCCTCCCCCCATAAC | Primers amplifying part of *RdRP4* |
| QDE3fg_F GCCGATCGGAGAGGAAGACAACGAACTCTT  QDE3rg_R GACACTACTTTGAAACACAGGCTGAGTGCC | Primers amplifying part of *QDE3* |
| RDR1_fg_F GGTGCTCCAAGCACCCAAACAACATATTTG  RDR1_rg_R TGGACTTGTGAGGAGGACGAGGGTGCCCA | Primers amplifying part of *RdRP1* |
| QIP_fg_F CACAAGGACTTTGCTTTCTGTCCGTTTAAG  QIP_rg_R GCATGCCCTGGGTGTCGATAGGCTCTCTCA | Primers amplifying part of *QIP* |
| FgAGO1_F TCCTCTCCAATTTCTTCCCCG  FgAGO1_R TGACTTCGACAGAACCGGAC | Primers amplifying part of *AGO1* |
| FgAGO2_F GGGATGGTGGCAAGTTCCTA  FgAGO2_R CTCCCTTTTGGATGTCGGCT | Primers amplifying part of *AGO2* |
| FgDCL1_F TGGTCTGCACTGCTCACATT  FgDCL1_R ATATTGCCAAGGGTGCTGCT | Primers amplifying part of *DCL1* |
| FgDCL2_F ACAAGCCCAATCTTTCCCGA  FgDCL2_R ATTCCCGAGCGTCGGATGA | Primers amplifying part of *DCL2* |
