# Supplementary material for: Different Components of the RNA Interference Machinery Are Required for Conidiation, Ascosporogenesis, Virulence, Deoxynivalenol Production, and Fungal Inhibition by Exogenous Double-Stranded RNA in the Head Blight Pathogen Fusarium graminearum
Source: Front Microbiol. 2019 Aug 7;10:1662. doi: 10.3389/fmicb.2019.01662 (PMC6764512; doi:10.3389/fmicb.2019.01662)
Supplement: Supplementary file 4 [file Table_3.docx]

**Table S3**. Primers used in qRT-PCR to check the relative mRNA expression of deleted genes.

| FgAGO2_F1 GCCAAGTTTGGTGAAGCTGG  FgAGO2_R1 CATGGGAATGAAGCCACCC | Forward and reverse primers for *AGO2* expression |
| --- | --- |
| AGO1qpcr_F1 TCCCAGTTTGGTCGGATTGG  AGO1qpcr_R1 TGCTGTCGGTTGTGCATTTG | Forward and reverse primers for *AGO1* expression |
| RDR1qpcr_F1 TATTGGAGGTCAGCTTGCGG  RDR1qpcr_R1 CTGCCTGTCGCTTCTGGTTA | Forward and reverse primers for *RdRP1* expression |
| QDE-3qpcr_F ATCCCTGACTCCGACGATGA  QDE-3qpcr_R AGCAGAGTCATCAACAACCAGT | Forward and reverse primers for *QDE3* expression |
| QIPqpcr_F2 AACAGATGGTTCCGCTGCAA  QIPqpcr_R CAAAGTCCTTGTGAACGGGC | Forward and reverse primers for *QIP* expression |
| DCL2qpcr_F3 TTGGAGGTTCAGCTCATCGC  DCL2qpcr_R3 TGACGATAGAGGGATGCGGA | Forward and reverse primers for *DCL2* expression |
| DCL1qpcr_F2 TGAGTCCCATTTCCAACAGCA  DCL1qpcr_R2 TTCCCTCCTCTGCCACTGAT | Forward and reverse primers for *DCL1* expression |
| RDR4qpcr1_F TGCCGAGAAAGCAACCTGAT  RDR4qpcr1_R GGAACAATGGTGCGCGATAC | Forward and reverse primers for *RdRP4* expression |
| RDR2qpcr1_F ACCACAACCAGACGAATGGA  RDR2qpcr1_R TGTCGTAGTTGATGACGGCAA | Forward and reverse primers for *RdRP2* expression |
| RDR3qpcr2_F GCGGCCTGTAAGTGGTATTG  RDR3qpcr2_R GGGCACCTCTCTTGTTCAGG | Forward and reverse primers for *RdRP3* expression |
| β-Tubulin_F ATCTCGAGCCCGGTACCATGG  β-Tubulin_R CTCGGTGTAATGACCCTTGGCC | Forward and reverse primers for Fusarium β-Tubulin reference gene |
| EF1a_F CAAGGCCGTCGAGAAGTCCAC  EF1a_R TGCCAACATGATCATTTCGTCGTA | Forward and reverse primers for Fusarium *Elongation Factor A* reference gene |
